# Who Were Hospitalized Deceased Patients from COVID-19 During the First Year of Pandemic? Retrospective Analysis of 1104 Deceased Patients in South of France

**DOI:** 10.1007/s44197-022-00039-3

**Published:** 2022-04-29

**Authors:** Sylvie Arlotto, Kevin Legueult, Alice Blin, Sebastien Cortaredona, Audrey Giraud-Gatineau, Laurent Bailly, Marie-Thérèse Jimeno, Léa Delorme, Philippe Brouqui, Jean-Christophe Lagier, Matthieu Million, Jean Dellamonica, Philippe Colson, Michel Carles, Didier Raoult, Christian Pradier, Stéphanie Gentile

**Affiliations:** 1 Aix Marseille Univ, School of Medicine-La Timone Medical Campus, EA 3279: CEReSS-Health Service Research and Quality of Life Center, Marseille, France; 2 Aix-Marseille Univ, IRD, APHM, MEPHI, Marseille, France; 3 Department of Public Health, Université Côte d’Azur, Centre Hospitalier Universitaire de Nice, Nice, France; 4 Aix Marseille Univ, Institut de Recherche pour le Développement (IRD), Assistance Publique-Hôpitaux de Marseille (AP-HM), Service de Santé des Armées (SSA), Vecteurs-Infections Tropicales et Méditerranéennes (VITROME), Marseille, France; 5 French Armed Forces Center for Epidemiology and Public Health (CESPA), Service de Santé des Armées (SSA), Marseille, France; 6 Assistance Publique-Hôpitaux de Marseille (AP-HM), Marseille, France; 7 Service d’Information Médicale Public Health Department La Timone Hospital, Marseille, France; 8 IHU-Méditerranée Infection, Marseille, France; 9 Intensive Care Unit, Université Côte d’Azur, UR2CA, Centre Hospitalier Universitaire de Nice, Nice, France; 10 Department of Infectious Diseases, Université Côte d’Azur, Centre Hospitalier Universitaire de Nice, Nice, France; 11 IHU Méditerranée Infection, Marseille, France; 12 Aix Marseille University, IRD, AP-HM, SSA, VITROME, Marseille, France

**Keywords:** COVID-19, SARS-CoV-2, Mortality, premature, Frailty, Comorbidity

## Abstract

**Introduction:**

Following the first year of the COVID-19 pandemic, a complete analysis of the characteristics of the deceased hospitalized patients was performed, to identify factors related to premature mortality and to compare patient profiles according to the epidemic periods.

**Methods:**

Retrospective analysis of 1104 deceased patients in two University Hospitals in South-eastern France, between March 1, 2020 and March 12, 2021 from Hospital’s electronic medical records was performed.

**Results:**

Mean age was 80 years (± 11.1) and 10% of the deceased were younger than 65 years with specific comorbidities, e.g., genetic conditions, metastatic cancer, or massive obesity. Among the three clusters identified, two clusters (75% of deceased patients) include very elderly patients with numerous comorbidities, and differ by their proportion of dependent institutionalized patients. The third cluster is made up of younger patients with fewer but severe comorbidities. Deceased patients’ profiles varied according to the epidemic periods: during the first period (March–June 2020), more patients were institutionalized. The second period (September–December2020) coincided with a higher mortality rate.

**Conclusions:**

This study confirmed that most patients hospitalized and dying from COVID-19 were frail, i.e., elderly and/or highly comorbid and that the small proportion of young patients had severe comorbidities.

**Supplementary Information:**

The online version contains supplementary material available at 10.1007/s44197-022-00039-3.

## Introduction

The World Health Organization (WHO) declared coronavirus disease 2019 (COVID-19) a pandemic on March 11, 2020 [1]. By March 12, 2021, COVID-19 had spread to 119 M people, resulting in 2.7 M deaths worldwide [2].

The first case of COVID-19 in France was diagnosed on January 24, 2020; by March 12, 2021, there were 4 M COVID-19 cases and 90,207 deaths.

As in the rest of Europe, France has experienced successive epidemic periods corresponding to different SARS-CoV-2 variants. French regions were not equally impacted during these different periods. Indeed, clear spatial heterogeneity of in-hospital COVID-19 incidence and mortality rates were found, following the spread of the epidemic according to various factors as well as population age structure, urban development and population density, economic level, health system, climatic and meteorological factors, and anti-contagion policies and practices [3]*.* Only two regions (Ile de France and Grand Est) had a higher mortality rate due to COVID-19 compared to other regions, but only during the first epidemic period [4]. The South-Eastern region of France was particularly affected, with 35,053 patients hospitalized for COVID-19, including 6140 deaths [5].

However, little is known of the medical and sociodemographic profile of deceased COVID-19 patients in Europe. As the available data are mainly derived from hospital information systems and/or death certificates, their quality is generally incomplete notably for sociodemographic characteristics. To our knowledge, no comprehensive study has focused on deceased COVID-19 patients in France, during the first year of pandemic.

Indeed, out of the 7 published studies that have investigated the profile of deceased patients, 5 are Chinese studies on the Wuhan epidemic [6–10], one is an Ethiopian study [11] and finally one study was conducted by an Italian team [12] All of these studies focused on the first months of epidemic, with limited study periods, from 1 to 2 months. Moreover, cohorts of COVID-19 deceased patients were small, between 14 and 320 patients.

However, all these studies already revealed that deceased patients were older and suffered more often from underlying comorbidities, particularly cardiovascular disease and diabetes. The Chinese studies focused mainly on the cause of death as well as on biological markers of disease severity.

Since then, other studies have investigated the risk factors for death in hospitalized COVID-19 patients and report that advanced age, as well as the presence of multiple comorbidities such as hypertension or diabetes, is uniform criteria regardless of the epidemic period and the associated variant [13–16].

For these reasons, the objective of this paper is to present a complete sociodemographic and clinical profile of patients who died in the two University Hospitals of South-eastern France, in Marseille and in Nice during the first year of COVID-19 pandemic. The secondary objectives were to identify the profiles related to premature mortality, i.e., before 65 years of age [17] and to compare deceased patients’ characteristics according to the different epidemic periods.

## Methods

### Design and Patient Selection

This was a retrospective analysis of all in-hospital deceased patients with a SARS-CoV-2 PCR-positive test in the University Hospitals of Marseille and Nice between March 1, 2020, and March 12, 2021. Among the 32 university hospital centers in France, only 2 are located in the Provence Alpes Côte d’Azur region: in Nice and Marseille. University hospitals are public institutions that are located in a major city and have highly specialized departments as well as research and teaching units. Then, they treat ordinary patients and COVID-19 patients and also have intensive care units.

Patients with positive PCR test but for whom death was not attributable to COVID-19 were excluded.

### Data Collection

We collected data from the medical records: sex, age, lifestyle profile, i.e., living environment, level of autonomy and bedridden state, the care pathway, i.e., circumstances of admission, transfer to the intensive care unit and the comorbidities.

All medical record data were collected anonymously.

*Patient and Public Involvement*: no patient was involved.

The number of hospitalizations of patients with COVID-19 was extracted from the hospital’s information system.

*Period of Outbreak*

We collected the date of the first positive PCR test for each patient to compare deceased patients’ profile according to the epidemic period [18]. The first epidemic period (Wuhan-Hu-1 SARS-CoV-2) lasted from March 1, 2020 to June 15, 2020; the second one from September 1, 2020 to December 31, 2020 (B.1.160 variant predominated) [19]; and the third one from January 2021 to the end of June 2021 (B.1.1.7 variant predominated) [20]. Variants were named according to the Pangolin classification [21].

In Marseille, a small outbreak between July and August 2020 involved a B.1.416 variant with few deaths which were excluded from the comparison [19, 22].

An illustration of the different COVID-19 epidemic period and predominant variants as a function of time in South-eastern France is available in supplementary Fig. SF1.

### Statistical Analysis

Age was grouped into the following classes: < 65 years; 65–74 years; 75–84 years; 85 years and over.

All data on deceased patients were compared between the different epidemic periods as defined above.

Categorical variables are presented as n (%) and continuous variables as mean (std) (or median and interquartile range in those with no criteria of normal distribution). We used the Wilcoxon Mann–Whitney test, Student’s *t* test, *χ*^2^ test, or Fisher’s exact test to compare differences between groups of patients.

To allocate patients to groups as suggested by the recorded data, we performed a multiple correspondence analysis (MCA) combined with an agglomerative hierarchical cluster analysis (AHCA) [23].

To identify factors specific to patients who died before age 65 (i.e., premature mortality), we performed a multivariable logistic regression. Two regression models were constructed, one with the obesity variable (BMI ≥ 30), the other with the massive obesity variable (BMI ≥ 40). Any statistically significant factor in univariate analyses (*p* < 0.1) was selected as a potential candidate for the multivariable logistic model.

All analyses involved two-sided *p* values, with statistical significance defined by *p* ≤ 0.05. Statistical analyses were performed with SPSS statistical software version 20. The MCA and AHCA were performed using the R 3.5.3 software and the FactomineR package [24].

## Results

**Fig. 1:**
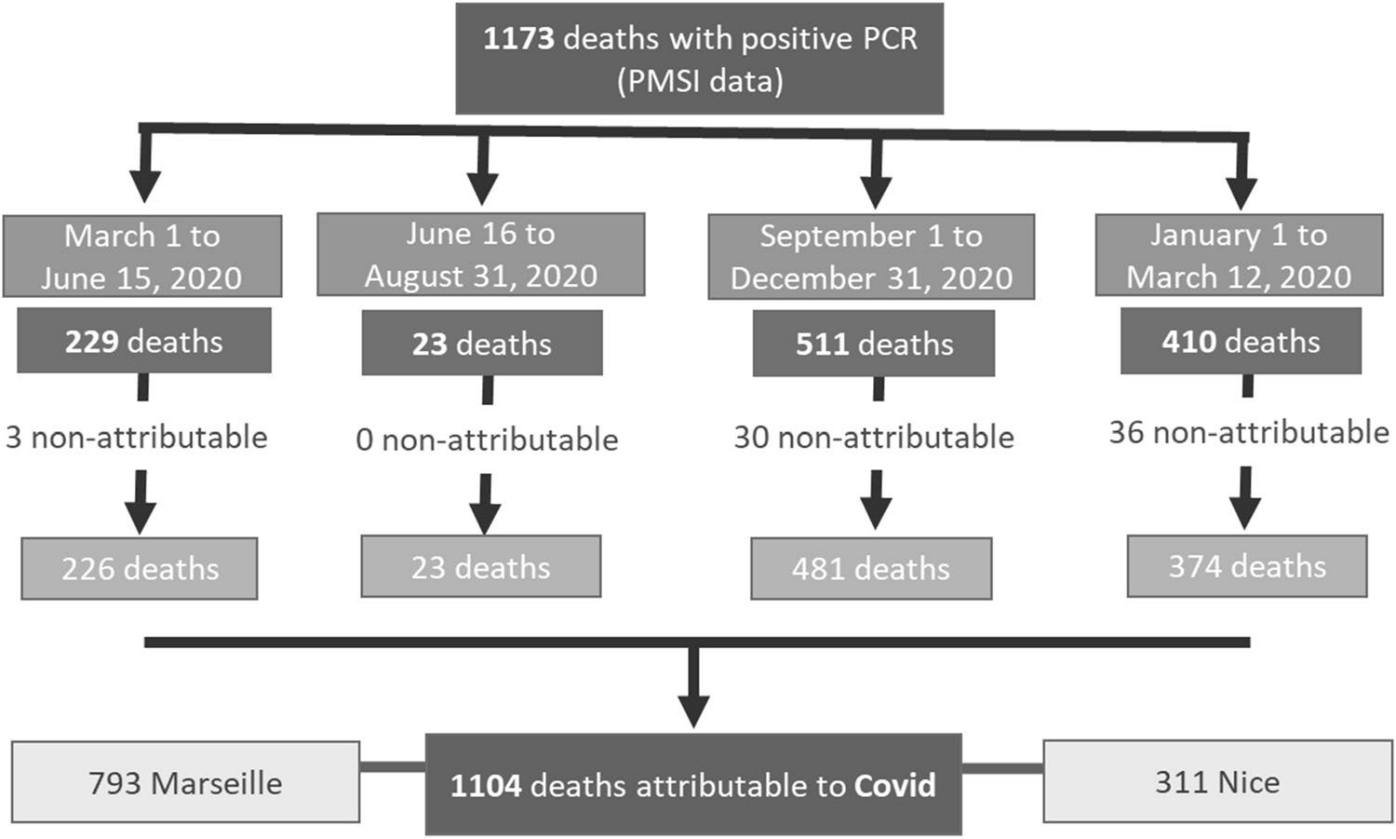
Flow chart

Between March 1, 2020 and March 12, 2021, 10,565 patients were hospitalized for COVID-19 in the University Hospitals of Marseille and Nice (7464 and 3101, respectively), of which 1173 died (793 and 311, respectively) (Fig. 1).

Of these deaths, 69 were not attributable to COVID-19 and were excluded. These had PCR-positive test but no related COVID-19 symptoms. Deaths were attributed to metastatic cancer (27 patients), trauma (11 patients), septic shock (9 patients), stroke (9 patients), cardiac decompensation (7 patients) or aspiration pneumonia (6 patients).

A total of 1104 (94%) deaths were, therefore, attributable to COVID-19, i.e., a mortality rate of 10.5%. (10% for Nice and 10.6% for Marseille).

All deceased patients’ characteristics for each of the two University Hospitals are presented in supplementary Table ST1.

The average age of the deceased patients was 80 years; the proportion of patients under 65 years of age was higher in Marseille. Two-thirds of deceased patients were men, and half of the deceased patients had loss of autonomy.

Two-thirds of the deceased patients were hypertensive, one-third were diabetic, and one in six was a patient with obesity. The prevalence of comorbidities was similar in both University Hospitals except for heart disorders, diabetes and genetic conditions. Deceased patients in Nice had a higher number of comorbidities. Of the 1104 patients who died from COVID-19, only 6 had no diagnosed comorbidity.

Over half (58%) of the deceased patients came from home, 21% from an institution and 21% from a previous hospitalization. Fewer deceased patients in Nice had been admitted in intensive care.

*Deceased Patient Clusters*

**Fig. 2:**
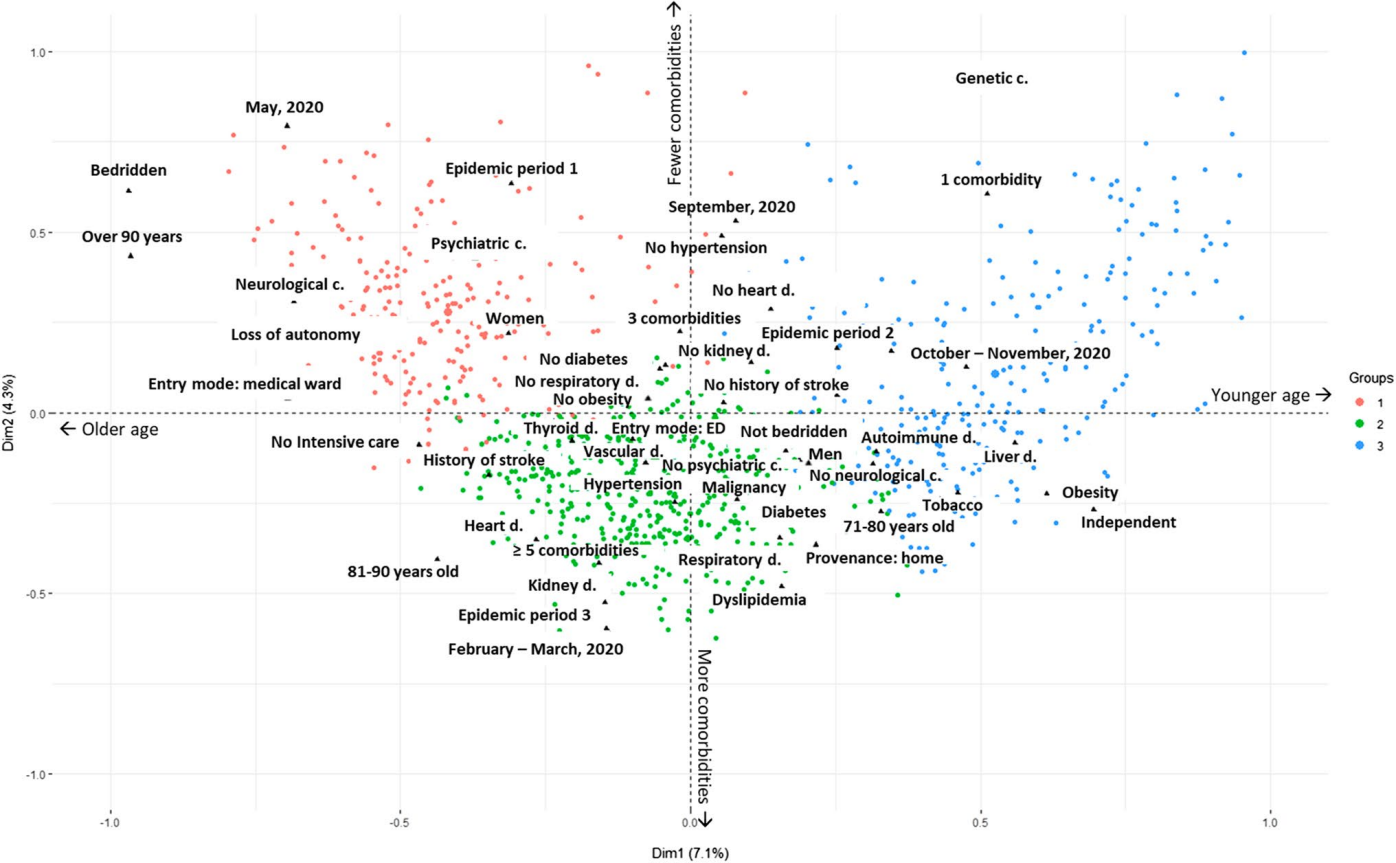
Three clusters of deceased patients

Three clusters of deceased patients were identified by AHCA (Fig. 2).

Each cluster includes patients with common sociodemographic characteristics and comorbidities. A comparison of the deceased COVID-19 patients’ characteristics according to the 3 clusters is presented in Table 1.

**Table 1:** Patients characteristics according to cluster defined by agglomerative hierarchical cluster analysis (*n* = 1104)

	Cluster 1	Cluster 2	Cluster 3	Total	*p*
Number of patients % (*n*)	23.8 (263)	51 (563)	25.2 (278)	(1104)	
Men % (*n*)	46 (121)	63.8 (359)	75.9 (211)	62.6 (691)	< 0.001
Age group % (*n*)					
0–40	0.4 (1)	0.0 (0)	0.7 (2)	0.3 (3)	< 0.001
41–50	0.8 (2)	0.0 (0)	3.2 (9)	1.0 (11)	
51–60	1.5 (4)	0.9 (5)	14 (39)	4.3 (48)	
61–70	7.6 (20)	2.7 (15)	40.3 (112)	13.3 (147)	
71–80	14.8 (39)	24.2 (136)	38.8 (108)	25.6 (283)	
81–90	42.6 (112)	57 (321)	2.9 (8)	39.9 (441)	
> 90	32.3 (85)	15.3 (86)	0.0 (0)	15.5 (171)	
Means age ± sd	84.9 ± 10.5	83.8 ± 7.1	67.7 ± 8.9	80 ± 11.1	< 0.001
Age under 65% (*n*)	4.6 (12)	1.8 (10)	29.9 (83)	9.5 (105)	< 0.001
Quality of life style data % (*n*)					
Loss of autonomy	100.0 (263)	49.4 (278)	14.0 (39)	52.5 (580)	< 0.001
Bedridden	43.3 (114)	6.6 (37)	3.2 (9)	14.5 (160)	< 0.001
Institutionalized	90.5 (238)	1.6 (9)	1.4 (4)	22.7 (251)	< 0.001
*Patient healthcare trajectory*					
Provenance % (*n*)					
Home	3.8 (10)	82.6 (465)	58.3 (162)	57.7 (637)	< 0.001
Institution	85.9 (226)	0.9 (5)	1.1 (3)	21.2 (234)	
Previous hospitalization	10.3 (27)	16.5 (93)	40.6 (113)	21.1 (233)	
Site of death % (*n*)					
Medical ward	82.5 (217)	80.8 (455)	16.9 (47)	65.1 (719)	< 0.001
Intensive care	0.8 (2)	9.2 (52)	82.7 (230)	25.7 (284)	
Emergency department	16.7 (44)	9.9 (56)	0.4 (1)	9.1 (101)	
Death in the first 24 h	17.9 (47)	7.8 (44)	5.8 (16)	9.7 (107)	< 0.001
Transfer to intensive care	0.4 (1)	8.7 (49)	85.3 (237)	26.0 (287)	< 0.001
Intensive care in the first 24 h	0.0 (0)	51.0 (25)	62.0 (147)	59.9 (172)	0.170
Epidemic period % (*n*)					
Epidemic period 1	36.1 (95)	14.4 (81)	18.0 (50)	20.5 (226)	< 0.001
Summer outbreak	1.9 (5)	2.3 (13)	1.8 (5)	2.1 (23)	
Epidemic period 2	39.9 (105)	39.1 (220)	56.1 (156)	43.6 (481)	
Epidemic period 3	22.1 (58)	44.2 (249)	24.1 (67)	33.9 (374)	
Comorbidities % (*n*)					
Active tumor	14.8 (39)	22.4 (126)	20.9 (58)	20.2 (223)	0.040
Metastasis	2.3 (6)	4.6 (26)	4.3 (12)	4 (44)	0.262
Heart disorder	43.7 (115)	50.1 (282)	30.6 (85)	43.7 (482)	< 0.001
Diabetes	21.7 (57)	32.3 (182)	38.5 (107)	31.3 (346)	< 0.001
Liver disease	3.4 (9)	1.6 (9)	9.7 (27)	4.1 (45)	< 0.001
Autoimmune disorder	3.8 (10)	5.7 (32)	8.3 (23)	5.9 (65)	0.084
Respiratory disease	17.1 (45)	27.7 (156)	29.5 (82)	25.6 (283)	0.001
Thyroid disorder	18.6 (49)	16.0 (90)	11.2 (31)	15.4 (170)	0.047
Vascular disease	24.3 (64)	20.8 (117)	22.7 (63)	22.1 (244)	0.501
History of stroke with or without hemiplegia	19.8 (52)	14.0 (79)	8.6 (24)	14.0 (155)	0.001
Neurological condition	67.3 (177)	25.4 (143)	10.1 (28)	31.5 (348)	< 0.001
Gastro-intestinal ulcer	5.7 (15)	9.1 (51)	5.0 (14)	7.2 (80)	0.058
Chronic kidney disease	14.4 (38)	17.1 (96)	9.7 (27)	14.6 (161)	0.018
Psychiatric condition	36.5 (96)	10.8 (61)	13.7 (38)	17.7 (195)	< 0.001
Genetic condition	3.8 (10)	1.2 (7)	5.4 (15)	2.9 (32)	0.002
Hypertension	58.6 (154)	72.8 (410)	60.8 (169)	66.4 (733)	< 0.001
Obesity	4.9 (13)	12.4 (70)	27.3 (76)	14.4 (159)	< 0.001
Massive obesity	0.0 (0)	0.7 (4)	5.4 (15)	1.7 (19)	< 0.001
Dyslipidemia	14.1 (37)	22.6 (127)	26.3 (73)	21.5 (237)	0.002
Tobacco	11.0 (29)	18.7 (105)	30.9 (86)	19.9 (220)	< 0.001
Alcohol	7.6 (20)	2.0 (11)	6.1 (17)	4.3 (48)	< 0.001
Average number of comorbidities ± sd	4.3 ± 1.9	4.1 ± 2.1	3.8 ± 2.2	4.1 ± 2.1	0.006
No comorbidity	0.0 (0)	0.0 (0)	2.2 (6)	0.5 (6)	< 0.001

Figure 2 displays an age gradient on the X axis and a comorbidity frequency gradient on the Y axis. Thus, elderly and comorbid patients are shown on the bottom left of the factorial plane and the young people without comorbidities on the top right.

The first cluster accounted for 23.8% of the deceased patients, (red dots in Fig. 2), mainly including institutionalized patients (90.5%). Most of the patients in this cluster were very elderly (84.9 years on average) and bedridden (43.3%), more than half of them were women (54%) while there were mostly men in the other two clusters. These patients were more likely to die within the first 24 h of admission (18%), and more often in the emergency department (16.7%). For these patients, the most prevalent comorbidities were neurological (67.3%) and psychiatric (36.5%) conditions.

The second cluster (in green) accounted for 50.9% of the deceased patients and mainly includes elderly patients (83.8 years on average) with loss of autonomy but not institutionalized and with several underlying conditions (4 comorbidities on average), e.g., active tumors, heart and respiratory disorders.

Finally, the third cluster accounted for 25.2% of the deceased patients (blue dots in Fig. 2). These patients were younger (67.7 years on average). They were more likely to be diabetic (38.5%), obese (27.3%), of which 19.7% with massive obesity, and to suffer from pre-existing respiratory disease (29.5%). They were more likely to smoke (30.9%). A small percentage had specific comorbidities: genetic conditions (5.4%), liver disease (9.7%) and autoimmune disorder (8.3%). Their clinical pathway was different: most of them were admitted to the intensive care (85.3%), 40.6% came from other hospitals in the region, as well as the 6 patients who died without diagnosed comorbidity. In this cluster, patients were more numerous during the third epidemic period.

*Characteristics of Deceased Patients According to Epidemic Periods*

The epidemic periods (P1, P2, and P3) differed in terms of duration and of number of cases and deaths. The hospital mortality rate was higher in P2. Deceased patients’ profile also varied between the different epidemic periods (Table ST2). During P1, there were more institutionalized patients among the deceased. During P2, deceased patients were younger and were more often transferred to an intensive care. Patients who died during the P3 had more heart disorders and more often hypertensive but had fewer neurological conditions.

*Specificities of Prematurely Deceased Patient Characteristics*

Of the 1104 deceased patients, 9.5% were under 65 years. Table 2 presents the characteristics of these deceased patients.

**Table 2:** Patient characteristics and risk of death according to age (105 deceased patients by premature mortality)

		Univariate analysis			Multivariable analysis First model		Multivariable analysis Second model	
	Total *N* = 1104	Age < 65 years *N* = 105	Age ≥ 65 years *N* = 999	*p*	Adjusted odds ratio (95% IC)	*p* value	Adjusted odds ratio (95% IC)	*p* value
Men % (*n*)	62.6 (691)	63.8 (67)	62.5 (624)	0.786				
Quality of life % (*n*)								
Loss of autonomy ***	52.5 (580)	31.4 (33)	54.8 (547)	< 0.001				
Bedridden	14.5 (160)	10.5 (11)	14.9 (149)	0.219				
Institutionalized ***	22.7 (251)	15.2 (16)	23.5 (235)	0.054				
*Patient healthcare trajectory*								
Provenance % (*n*)								
Home	57.7 (637)	49.5 (52)	58.6 (585)	< 0.001				
Institution	21.2 (234)	13.3 (14)	22.0 (220)					
Previous hospitalization**	21.1 (233)	37.1 (39)	19.4 (194)		1.95 (1.20–3.17)	0.007	2.01 (1.23–3.29)	0.005
Entry mode *** % (*n*)								
Emergency department	75.0 (828)	66.7 (70)	75.9 (758)	< 0.001				
Medical ward	19.4 (214)	15.2 (16)	19.8 (198)					
Intensive care	5.6 (62)	18.1 (19)	4.3 (43)					
Site of death *** % (*n*)								
Medical ward	65.1 (719)	36.2 (38)	68.2 (681)	< 0.001				
Intensive care	25.7 (284)	61.9 (65)	21.9 (219)					
Emergency department	9.1 (101)	1.9 (2)	9.9 (99)					
Death within the first 24 h	9.7 (107)	8.6 (9)	9.8 (98)	0.683				
Transfer to intensive care*	26.0 (287)	65.7 (69)	21.8 (218)	< 0.001	6.30 (3.91—10.17)	< 0.001	6.15 (3.81 – 9.93)	< 0.001
Intensive care within the first 24 h	59.9 (172)	62.3 (43)	59.2 (129)	0.642				
Comorbidities % (*n*)								
Active tumor	20.2 (223)	23.8 (25)	19.8 (198)	0.333				
Metastasis*	4.0 (44)	8.6 (9)	3.5 (35)	0.030	3.74 (1.53 –9.15)	0.004	3.59 (1.48–8.76)	0.005
Heart disorder *	43.7 (482)	21.0 (22)	46.0 (460)	< 0.001	0.42 (0.25–0.71)	0.001	0.42 (0.25–0.72)	0.002
Diabetes	31.3 (346)	27.6 (29)	31.7 (317)	0.387				
Liver disease*	4.1 (45)	9.5 (10)	3.5 (35)	0.007				
Autoimmune disorder*	5.9 (65)	11.4 (12)	5.3 (53)	0.011				
Respiratory disease*	25.6 (283)	33.3 (35)	24.8 (248)	0.057				
Sleep apnea***	8.1 (89)	15.2 (16)	7.3 (73)	0.005				
Asthma	4.5 (50)	4.8 (5)	4.5 (45)	0.807				
Thyroid disorder	15.4 (170)	10.5 (11)	15.9 (159)	0.142				
Vascular disease	22.1 (244)	21.9 (23)	22.1 (221)	0.959				
History of stroke with or without hemiplegia*	14.0 (155)	7.6 (8)	14.7 (147)	0.046				
Neurological condition***	31.5 (348)	17.1 (18)	33.0 (330)	0.001				
Gastro-intestinal ulcer	7.2 (80)	9.5 (10)	7.0 (70)	0.344				
Chronic kidney disease	14.6 (161)	12.4 (13)	14.8 (148)	0.501				
Psychiatric condition	17.7 (195)	21.0 (22)	17.3 (173)	0.353				
Genetic condition*	2.9 (32)	12.4 (13)	1.9 (19)	< 0.001	6.09 (2.52–14.72)	< 0.001	6.10 (2.51–14.80)	< 0.001
Hypertension*	66.4 (733)	40.0 (42)	69.2 (691)	< 0.001	0.28 (0.17 –0.45)	< 0.001	0.29 (0.18–0.45)	< 0.001
Obesity*^1^	14.4 (159)	21.9 (23)	13.6 (136)	0.021				
Massive obesity*^2^	1.7 (19)	6.7 (7)	1.2 (12)	0.001			3.36 (1.10–10.32)	0.034
Dyslipidemia	21.5 (237)	15.2 (16)	22.1 (221)	0.102				
Tobacco***	19.9 (220)	29.5 (31)	18.9 (189)	0.010				
Alcohol	4.3 (48)	7.6 (8)	4.0 (40)	0.124				
Average number of total comorbidities ± sd***	4.1 ± 2.1	3.5 ± 2.3	4.2 ± 2	0.003				
Min−max (median)	0–12 (4)	0–10 (3)	0–12 (4)					
No comorbidity ***	0.5 (6)	4.8 (5)	0.1 (1)	< 0.001				

*Variables entered in the multivariable analysis. (*^1^ variables entered in the first model, *^2^ variables entered in the second model)

**Variables entered in the multivariable analysis after binarization

***Variables not entered in the multivariable analysis because highly correlated with other covariate(s)

Among these 105 who were under 65, 21 had fewer than two comorbidities (1.9% of all deceased patients): 16 were male and only 2 patients were under 40 years. All had been admitted to the intensive care, except for two terminally ill patients with a condition other than COVID-19. Among the patients admitted to the intensive care, half of them were admitted within the first 24 h of hospitalization.

Two multivariate analysis models were performed to identify the characteristics of these younger patients (Table 2). The first model, using BMI > 30, showed that they were 6 times more likely to suffer from a genetic condition and 4 times more likely to have a metastatic cancer and to be hospitalized in intensive care. The second model, using BMI ≥ 40, yielded the same factors but found that patients who died before the age of 65 were 3 times more likely to have massive obesity.

## Discussion

Our study shows that 90% of deceased patients during the first year of the epidemic were at least 65 years old. In addition to their very poor state of health related to age, most of them suffered from severe chronic conditions. This finding is consistent with data already published in the literature showing that the risk of death due to COVID-19 strongly depends on patients’ age and previous health status [6–11, 25].

Among the three distinct clusters, the first two (3/4 of the deceased patients) are very old and comorbid patients: one of the two cluster is represented by institutionalized patients, often bedridden and demented, with neurological and/or psychiatric conditions; the second concerns elderly patients living at home who have lost their autonomy. These elderly patients suffer from heart disorders, hypertension, respiratory diseases, diabetes and malignancy, according to literature review [6, 12, 26–30]. They are, therefore, frail patients who were particularly at risk of dying from an acute event such as COVID-19 [31], a situation already described during influenza epidemics [32], supporting the recommendation to target these populations for vaccination.

Finally, the third cluster includes younger patients, with fewer comorbidities but severe, i.e., genetic diseases and metastatic cancers, and/or massive obesity, testifying to their frailty, who are opportunely targeted by current vaccination campaigns [33].

In our study, deceased patients under 65 years of age represented 10%, similar to that reported by Biagi et al. [12].

Only 2% of patients under 65 years of age had fewer than two comorbidities, suggesting potentially undiagnosed comorbidities. Several articles already focused on this subject and showed that the infection appears to be more severe in certain patients, suggesting that genetic or epigenetic factors are at play [34]. Surprisingly, although univariate analysis revealed that patients younger than 65 years had more autoimmune and liver disease, multivariate analysis did not identify these factors as being related to premature mortality. In addition, although patients younger than 65 years were significantly more obese, the multivariate analysis did not identify obesity (BMI > 30) as a factor related to premature mortality. Obesity (BMI > 30) and autoimmune and liver disease appear to be a factor in hospitalization and severity, but not necessarily a factor related to premature mortality.

Since the elderly are those most at risk of death, the role of geriatricians in the management of these patients should be considered. In the literature, several authors deplore the fact that the context of a health emergency has given rise only to acute management without multidisciplinary collaboration including geriatricians. Collaboration with geriatric physicians is always valuable, especially since the clinical and biological expression of disease in the elderly is different, often presenting as confusion, falls or diarrhea [35], as is their tolerance to treatment, notably to corticosteroids [36]. In addition, the isolation to which the elderly were exposed during the pandemic and the resulting loneliness may have increased their physical and psychological frailty [37]. If the epidemics of COVID-19 were to continue, frailty should be addressed to provide the most effective therapy and multidisciplinary and geriatric management should be anticipated [36]. In addition, to avoid the negative consequences of isolation and loneliness, multicomponent programs with adequate strategies [38].

The analysis of the profile of the deceased patients according to the different epidemic periods shows some differences in profile even if globally the elderly and with comorbidities subject remains the first victim. During the first epidemic period, we observed a higher proportion of patients coming from institutions for elderly people. This can be explained by the lack of knowledge of the disease, the lack of means to test patients and of personal protective equipment in addition to the lockdown of France during this first period, which led to the fact that only public hospitals were able to take care of patients with COVID-19.

The second epidemic period lasted much longer and was more deadly. The mortality rate of hospitalized patients was also higher and the proportion of young patients was higher as well the rate of admission to intensive care, despite improved medical management and better knowledge of COVID-19 disease. These results show the virulence of this variant [39]. In view of such variation, epidemiological surveillance of deceased patient should continue to better anticipate a possible change of target.

A limitation of this study is that our analysis focused on deaths in two reference hospitals for complex cases requiring high-level resuscitation, which certainly led to overestimate the proportion of “young” subjects. Our patients represent only 18% of all deceased patients hospitalized in our region [40].

As the peak of the third epidemic period was reached on April 12, 2021 and the epidemic ended at the end of June 2021 [20], we cannot conclude on the impact of the B.1.1.7 variant on the profile of deceased patients. Since the end of this work, a fourth period has taken place in France with a new variant (B.1.617.2). The data on the profile of the patients are not known.

## Conclusions

This study confirms that most patients hospitalized and dying from COVID-19 were elderly and/or highly comorbid. In addition, premature mortality concerns 10% of our population of deceased patients, with specific profiles, e.g., autoimmune and genetic conditions.

This reinforces the arguments for the vaccine strategy including these at-risk populations as a priority and suggests the need for multidisciplinary care, involving a geriatrician, for elderly and frail patients.

## Supplementary Information

Below is the link to the electronic supplementary material.

Supplementary file1 (DOCX 17 KB)

Supplementary file2 (PDF 483 KB)

Supplementary file3 (DOCX 16 KB)

## Data Availability

The datasets used and/or analyzed during the current study are available from the corresponding author on reasonable request.

## Abbreviations

MCA: Multiple correspondence analysis
AHCA: Agglomerative hierarchical cluster analysis
P1: First epidemic period
P1: Second epidemic period
P1: Third epidemic period
