# Supplementary material for: Who Were Hospitalized Deceased Patients from COVID-19 During the First Year of Pandemic? Retrospective Analysis of 1104 Deceased Patients in South of France
Source: J Epidemiol Glob Health. 2022 Apr 29;12(2):196–205. doi: 10.1007/s44197-022-00039-3 (PMC9053122; doi:10.1007/s44197-022-00039-3)
Supplement: Supplementary file 1 — Supplementary file1 (DOCX 17 KB) [file 44197_2022_39_MOESM1_ESM.docx]

Supplementary Table 2_ Comparative table of epidemic periods (n = 1,081).

|  | **Epidemic period 1**  **N = 226** | **Epidemic period 2**  **N = 481** | **Epidemic period 3**  **N = 374** | **Total**  **N= 1081** | **p** |
| --- | --- | --- | --- | --- | --- |
| Men % (n) | 59.7 (135) | 66.3 (319) | 59.1 (221) | 62.4 (675) | .061 |
| **Age Group % (n)** |  |  |  |  |  |
| 0-40 | 0.4 (1) | 0.0 (0) | 0.5 (2) | 0.3 (3) | .119 |
| 41-50 | 0.9 (2) | 1.2 (6) | 0.8 (3) | 1.0 (11) |  |
| 51-60 | 4.9 (11) | 5.4 (26) | 2.7 (10) | 4.3 (47) |  |
| 61-70 | 10.6 (24) | 15.4 (74) | 12.3 (46) | 13.3 (144) |  |
| 71-80 | 28.3 (64) | 27.4 (132) | 21.9 (82) | 25.7 (278) |  |
| 81-90 | 39.4 (89) | 36.6 (176) | 44.1 (165) | 39.8 (430) |  |
| > 90 | 15.5 (35) | 13.9 (67) | 17.6 (66) | 15.5 (168) |  |
| Means age ± sd | 80.1 ± 11.3 | 79 ± 11 | 81.2 ± 11 | 80 ± 11.1 | .021 |
| Age under 65 % (n) | 8,8 (20) | 10,6 (51) | 8,8 (33) | 9,6 (104) | .618 |
| **City of inclusion % (n)** |  |  |  |  |  |
| Marseille | 78.8 (178) | 76.7 (369) | 60.2 (225) | 71.4 (772) | < .001 |
| Nice | 21.2 (48) | 23.3 (112) | 39.8 (149) | 28.0 (309) |  |
| **Quality of life style data % (n)** |  |  |  |  |  |
| Loss of autonomy | 63.7 (144) | 48.6 (234) | 51.6 (193) | 52.8 (571) | .001 |
| Bedridden | 21.7 (49) | 13.1 (63) | 11.8 (44) | 14.4 (156) | .002 |
| Institutionalized | 38.1 (86) | 20.6 (99) | 16.3 (61) | 22.8 (246) | < .001 |
| **Patient healthcare trajectory** |  |  |  |  |  |
| **Provenance % (n)** |  |  |  |  |  |
| Home | 50.0 (113) | 57.0 (274) | 62.6 (234) | 57.4 (621) | < .001 |
| Institution | 34.5 (78) | 19.8 (95) | 15.2 (57) | 21.3 (230) |  |
| Previous hospitalization | 15.5 (35) | 23.3 (112) | 22.2 (83) | 21.3 (230) |  |
| **Site of death % (n)** |  |  |  |  |  |
| Medical ward | 71.2 (161) | 60.7 (292) | 66.8 (250) | 65.0 (703) | < .001 |
| Intensive care | 22.1 (50) | 32.8 (158) | 18.7 (70) | 25.7 (278) |  |
| Emergency department | 6.6 (15) | 6.4 (31) | 14.4 (54) | 9.3 (100) |  |
| Death in the first 24 hours | 10.6 (24) | 8.5 (41) | 10.2 (38) | 9.5 (103) | .592 |
| Transfer to intensive care | 23.5 (53) | 32.2 (155) | 19.3 (72) | 25.9 (280) | < .001 |
| Intensive care in the first 24 hours | 52.8 (28) | 66.5 (103) | 51.4 (37) | 60.0 (168) | .049 |
| **Comorbidities % (n)** |  |  |  |  |  |
| Active tumor | 17.7 (40) | 20.2 (97) | 21.7 (81) | 20.2 (218) | .504 |
| Metastasis | 4.9 (11) | 2.9 (14) | 4.5 (17) | 3.9 (42) | .326 |
| Heart disorder | 37.6 (85) | 41.4 (199) | 50.8 (190) | 43.8 (474) | .002 |
| Diabetes | 30.1 (68) | 31.8 (153) | 31.3 (117) | 31.3 (338) | .900 |
| Liver disease | 2.7 (6) | 3.5 (17) | 5.6 (21) | 4.1 (44) | .150 |
| Autoimmune disorder | 2.7 (6) | 7.7 (37) | 5.9 (22) | 6.0 (65) | .031 |
| Respiratory disease | 25.7 (58) | 22.2 (107) | 29.1 (109) | 25.3 (274) | .070 |
| Thyroid disorder | 17.7 (40) | 14.8 (71) | 15.2 (57) | 15.5 (168) | .591 |
| Vascular disease | 25.3 (53) | 21.8 (105) | 21.9 (82) | 22.2 (240) | .878 |
| History of stroke with or without hemiplegia | 15.9 (36) | 14.1 (68) | 13.4 (50) | 14.2 (154) | .682 |
| Neurological condition | 39.4 (89) | 30.6 (147) | 28.1 (105) | 31.5 (341) | .013 |
| Gastro-intestinaI ulcer | 6.6 (15) | 7.5 (36) | 7.5 (28) | 7.3 (79) | .909 |
| Chronic kidney disease | 11.1 (25) | 14.1 (68) | 16.8 (63) | 14.4 (156) | .144 |
| Psychiatric condition | 20.4(46) | 19.1 (92) | 14.4 (54) | 17.8 (192) | .106 |
| Genetic condition | 2.7 (6) | 2.7 (13) | 3.5 (13) | 3.0 (32) | .767 |
| Hypertension | 61.5 (139) | 64.9 (312) | 71.1 (266) | 66.3 (717) | .036 |
| Obesity | 11.5 (26) | 14.1 (68) | 16.3 (61) | 14.3 (155) | .262 |
| Massive obesity | 1.3 (3) | 1.9 (9) | 1.9 (7) | 1.8 (19) | .858 |
| Dyslipidemia | 19.5 (44) | 22.2 (107) | 22.2 (83) | 21.6 (234) | .671 |
| Tobacco | 19.9 (45) | 19.8 (95) | 20.9 (78) | 20.2 (218) | .918 |
| Alcohol | 4.0 (9) | 3.1 (15) | 6.4 (24) | 4.4 (48) | .063 |
| **Average number of comorbidities** ± sd | 4 ± 2.2 | 4 ± 2 | 4.3 ± 2.1 | 4.1 ± 2.1 | .057 |
| **No comorbidity** | 0.9 (2) | 0.8 (4) | 0.0 (0) | 0.6 (6) | .202 |
