# Supplementary figures and images for: Who Were Hospitalized Deceased Patients from COVID-19 During the First Year of Pandemic? Retrospective Analysis of 1104 Deceased Patients in South of France
Source: J Epidemiol Glob Health. 2022 Apr 29;12(2):196–205. doi: 10.1007/s44197-022-00039-3 (PMC9053122; doi:10.1007/s44197-022-00039-3)

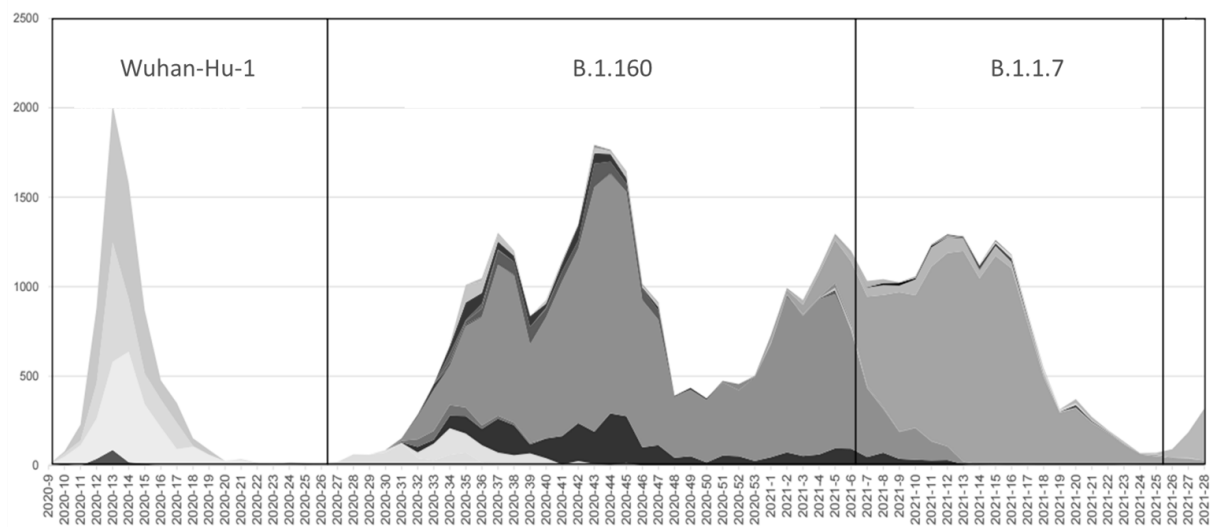

Supplementary Figure 1 – Epidemic periods and associate COVID-19 variants

Supplement: Supplementary file 2 — Supplementary file2 (PDF 483 KB) [file 44197_2022_39_MOESM2_ESM.pdf]
