# Supplementary material for: Who Were Hospitalized Deceased Patients from COVID-19 During the First Year of Pandemic? Retrospective Analysis of 1104 Deceased Patients in South of France
Source: J Epidemiol Glob Health. 2022 Apr 29;12(2):196–205. doi: 10.1007/s44197-022-00039-3 (PMC9053122; doi:10.1007/s44197-022-00039-3)
Supplement: Supplementary file 3 — Supplementary file3 (DOCX 16 KB) [file 44197_2022_39_MOESM3_ESM.docx]

Supplementary Table 1_ Characteristics of patients according to University Hospital (n = 1,104)

|  | **MARSEILLE** | **NICE** | **Total** | **p** |
| --- | --- | --- | --- | --- |
| **Number of patients** % (n) | 71.8 (793) | 28.2 (311) | 100.0 (1104) |  |
| Men % (n) | 62.9 (499) | 61.7 (192) | 62.6 (691) | .713 |
| **Age Group % (n)** |  |  |  |  |
| 0-40 | 0.4 (3) | 0.0 (0) | 0.3 (3) |  |
| 41-50 | 1.1 (9) | 0.6 (2) | 1.0 (11) |  |
| 51-60 | 5.2 (41) | 2.3 (7) | 4.3 (48) |  |
| 61-70 | 15.9 (126) | 6.8 (21) | 13.3 (147) | < .001 |
| 71-80 | 25.1 (199) | 27.0 (84) | 25.6 (283) |  |
| 81-90 | 38.8 (308) | 42.8 (133) | 39.9 (441) |  |
| > 90 | 13.5 (107) | 20.6 (64) | 15.5 (171) |  |
| Means age ± sd | 79 ± 11.4 | 82.6 ± 9.6 | 80 ± 11.1 | < .001 |
| Age under 65 years % (n) | 11.8 (92) | 4.2 (13) | 9.5 (105) | < .001 |
| **Quality of life style data % (n)** |  |  |  |  |
| Loss of autonomy | 53.1 (421) | 51.1 (159) | 52.5 (580) | .557 |
| Bedridden | 15.0 (119) | 13.2 (41) | 14.5 (160) | .439 |
| Institutionalized | 22.8 (181) | 22.5 (70) | 22.7 (251) | .910 |
| **Patient healthcare trajectory** |  |  |  |  |
| **Provenance % (n)** |  |  |  |  |
| Home | 57.0 (452) | 59.5 (185) | 57.7 (637) |  |
| Institution | 21.6 (171) | 20.3 (63) | 21.2 (234) | .753 |
| Previous hospitalization | 21.4 (170) | 20.3 (63) | 21.1 (233) |  |
| **Site of death % (n)** |  |  |  |  |
| Medical ward | 68.2 (541) | 57.2 (178) | 65.1 (719) |  |
| Intensive care | 27.6 (219) | 20.9 (65) | 25.7 (284) | < .001 |
| Emergency department | 4.2 (33) | 21.9 (68) | 9.1 (101) |  |
| Death in the first 24 hours | 8.8 (70) | 11.9 (37) | 9.7 (107) | .121 |
| Transfer to intensive care | 29.3 (232) | 17.7 (55) | 26.0 (287) | < .001 |
| Intensive care in the first 24 hours | 60.3 (140) | 58.2 ( 32) | 59.9 (172) | .769 |
| **Comorbidities % (n)** |  |  |  |  |
| Active tumor | 19.9 (158) | 20.9 (65) | 20.2 (223) | .716 |
| Metastasis | 4.2 (33) | 3.5 (11) | 4.0 (44) | .633 |
| Heart disorder | 41.2 (327) | 49.8 (155) | 43.7 (482) | .010 |
| Diabetes | 33.4 (265) | 26.0 (81) | 31.3 (346) | .018 |
| Liver disease | 4.7 (37) | 2.6 (8) | 4.1 (45) | .114 |
| Autoimmune disorder | 6.2 (49) | 5.1 (16) | 5.9 (65) | .511 |
| Respiratory disease | 25.3 (201) | 26.4 (82) | 25.6 (283) | .727 |
| Thyroid disorder | 15.4 (122) | 15.4 (48) | 15.4 (170) | .984 |
| Vascular disease | 22.1 (175) | 22.2 (69) | 22.1 (244) | .966 |
| History of stroke with or without hemiplegia | 12.9 (102) | 17.0 (53) | 14.0 (155) | .072 |
| Neurological condition | 30.4 (241) | 34.4 (107) | 31.5 (348) | .197 |
| Gastro-intestinaI ulcer | 3.6 (50) | 9.6 (30) | 7.2 (80) | .054 |
| Chronic kidney disease | 14.9 (118) | 13.8 (43) | 14.6 (161) | .655 |
| Psychiatric condition | 17.7 (140) | 17.7 (55) | 17.7 (195) | .990 |
| Genetic condition | 3.8 (30) | 0.6 (2) | 2.9 (32) | .005 |
| Hypertension | 67.8 (538) | 62.7 (195) | 66.4 (733) | .104 |
| Obesity | 14.4 (114) | 14.5 (45) | 14.4 (159) | .968 |
| Massive obesity | 1.8 (14) | 1.6 (5) | 1.7 (19) | .856 |
| Dyslipidemia | 20.2 (160) | 24.8 (77) | 21.5 (237) | .095 |
| Tobacco | 25.2 (200) | 6.4 (20) | 19.9 (220) | < .001 |
| Alcohol | 4.9 (39) | 2.9 (9) | 4.3 (48) | .138 |
| **Average number of comorbidities** ± sd | 3.97 ± 2.1 | 4.39 ± 2.1 | 4.09 ± 2.1 | .002 |
| **No comorbidity** | 0.8 (6) | 0.0 (0) | 0.5 (6) | .124 |
